# Retinal thinning in amyotrophic lateral sclerosis patients without ophthalmic disease

**DOI:** 10.1371/journal.pone.0185242

**Published:** 2017-09-25

**Authors:** Nisha Mukherjee, Shan McBurney-Lin, Anthony Kuo, Richard Bedlack, Henry Tseng

**Affiliations:** 1 Department of Ophthalmology, Duke University Medical Center, Durham, North Carolina, United States of America; 2 Duke University School of Medicine, Durham, North Carolina, United States of America; 3 Duke ALS Clinic, Duke University Medical Center, Durham, North Carolina, United States of America; Bascom Palmer Eye Institute, UNITED STATES

## Abstract

**Importance:**

Amyotrophic lateral sclerosis (ALS) is a fatal, rapidly progressive neurodegenerative disease that primarily affects motor neurons. Recently, three causative genes have been implicated in both ALS and glaucoma. However, it is still uncertain whether patients with ALS have neurodegeneration in their retinas. If so, retinal thickness measurements might be a useful biomarker for ALS progression. Previous work in this area has been inconclusive, as it has not taken into account the effect of ophthalmic diseases on retinal thinning.

**Objective:**

To determine whether there are differences in retinal neurons in ALS patients utilizing spectral-domain optical coherence tomography (SD-OCT). We tested the hypothesis that ALS patients exhibit retinal neurodegeneration that is not associated with ophthalmic diseases.

**Design, settings and participants:**

Observational, comparative, cross-sectional study performed on patients recruited from the Duke University Medical Center ALS clinic. Patients underwent a comprehensive ophthalmologic examination to rule out ocular pathology. 21 patients met inclusion criteria. Two eyes with ocular pathology were excluded, leading to a total of 40 eyes of 21 patients included in the study. Retinal neurodegeneration was assessed by retinal nerve fiber layer (RNFL) thickness measurement using SD-OCT (Spectralis; Heidelberg Engineering).

**Main outcomes and measures:**

ALS disease severity, determined through the ALS Functional Rating Scale (ALSFRS-R); mean and six sector RNFL thickness values compared to age-adjusted values in the normative database provided by Heidelberg Engineering; RNFL thickness correlation with ALSFRS-R, ALSFRS-R progression rate, forced vital capacity (FVC), and visual acuity.

**Results:**

ALSFRS-R mean score was 30+/-10. Mean RNFL thickness in ALS patients was 88.95 +/- 10.8 microns, significantly thinner than values in the normative database (95.81 +/- 0.8). These RNFL thickness values did not demonstrate correlation to ALSFRS-R score, ALSFRS-R progression rate, FVC, intraocular pressure, or visual acuity.

**Conclusions:**

Using SD-OCT, our study shows that ALS patients without ocular pathology exhibit thinned retinal layers. Future studies are warranted to clarify the clinical relationship between retinal thinning and motor neuron loss in ALS.

## Introduction

Amyotrophic lateral sclerosis (ALS) is a progressive neurodegenerative disorder of upper and lower motor neurons, resulting in death within three years of symptom onset in up to 70% of patients [1]. Though the molecular mechanism remains to be elucidated, motor neuron degeneration has been linked to various biochemical pathways, including oxidative stress, mitochondrial damage, neurofilament defects, and defects in RNA processing [2]. Recently, genetic associations were unexpectedly established between ALS and glaucoma, a disease characterized by blindness as a result of retinal ganglion cell degeneration. Three genes—optineurin, TBK1, and ataxin2—are associated with both familial ALS [3–6] and glaucoma [7–9], and the protein products for optineurin and TBK1 directly interact with one another [10]. These surprising findings suggest that ALS and glaucoma may share a common molecular mechanism. Alternatively, retinal neurons may be affected by the same neurodegenerative process as motor neurons in ALS, and may demonstrate degenerative changes even in the absence of glaucoma or other ophthalmic disease. If true, we hypothesize that retinas would be thinner in ALS patients.

Because the eye is the only anatomical location where CNS neurons can be directly examined and imaged by tools with higher resolution than current neuroimaging techniques—such as MRI and CT—there are increasing efforts to use the retina to diagnose neurological diseases. For example, a new clinical diagnostic method under development can directly image individual retinal ganglion cells that are undergoing apoptosis [11]. However, relatively little is known about retinal alterations upon clinical examination of ALS patients. Performing a detailed ophthalmic examination in ALS patients can be challenging in the eye clinic, due to difficulty in positioning them in the examination chair and the slit lamp biomicroscope. Many ALS patients are quickly fatigued and are unable to tolerate the full duration of the examination, including detailed refraction or the 20–30 minutes required for pupillary dilation to facilitate an examination of the retina and optic nerve. Furthermore, ALS patients with advanced disease may have dyspnea, difficulty with secretions, weakness, or spasticity, limiting their ability to travel to an outpatient ophthalmic clinic. Due to these issues, detailed ophthalmic examinations are often unable to be included as part of the medical care and treatment plans for ALS patients.

The development of optical coherence tomography (OCT) has allowed relatively quick and non-invasive evaluation of neurodegenerative patients in the clinic [12–14]. When used in conjunction with standard ophthalmological examination techniques, such as slit lamp biomicroscopy, OCT imaging can be completed within minutes and often without pupillary dilation. Spectral domain OCT (SD-OCT) provides higher resolution images of retinal cross-sections, which allows a more accurate measurement of the thickness of retinal layers. Because loss of retinal neurons is associated with retinal thinning, a decrease in the retinal nerve fiber layer (RNFL) adjacent to the optic nerve has been used as a marker of retinal ganglion cell loss [12–14]. Peripapillary RNFL thinning as measured through SD-OCT has been observed in neurodegenerative diseases such as Parkinson’s disease, Alzheimer’s disease, and multiple sclerosis [15–22].

Previous studies have utilized OCT technology to study eyes of ALS patients; however, they have been contradictory and thus inconclusive regarding whether there is actual retinal thinning [23–26]. This discrepancy is likely attributed to confounding clinical factors that were not excluded, such as ophthalmic diseases that may directly result in retinal changes and degeneration. Therefore, to test our hypothesis that ALS patients exhibit retinal thinning, we performed a study of ALS patients in which ocular medical diseases were carefully excluded by history and a comprehensive ophthalmic examination. Finally, we utilized the ALS Functional Rating Scale (ALSFRS-R) score as a measure of disease severity [27] to correlate ALS disease severity with ophthalmic changes that were not attributable to ocular disease.

## Materials and methods

The study was approved by the institutional review board of Duke University Medical Center and was performed in a HIPAA-compliant manner. This research adhered to the tenets of the Declaration of Helsinki. Patients were recruited from the Duke ALS clinic between 2013 and 2014. Patients who were medically unstable or had an established diagnosis of an ocular disease such as macular disease that could confound OCT measurements were not enrolled. The exclusion criteria also included any patients with a diagnosis of bilateral retinal diseases, glaucoma, or other optic neuropathies.

### Subjects

23 patients with ALS were enrolled for this study after written informed consent was obtained from each patient or legally authorized representative. Eligible patients were identified by clinical staff with a confirmed diagnosis of ALS made by a neurologist specializing in ALS, who also established the ALSFRS-R score for each patient [27]. From the 23 enrolled subjects, two were excluded because they could not be positioned in the sitting position to obtain standard SD-OCT imaging. From the remaining 21 subjects, one eye with a history of a retinal detachment and one eye with a history of a macular hole were excluded from analysis.

### Assessment

All qualified subjects underwent ophthalmologic evaluation that included visual acuity testing, intraocular pressure measurement by Tono-Pen, anterior segment examination, and undilated retinal examination of the optic nerve and posterior pole. For 20/21 participants, visual acuity was recorded using a Snellen visual acuity chart and results were converted to the log of the minimal angle of resolution (logMAR) to facilitate statistical analysis. We were unable to measure visual acuity for the remaining one subject due to inability to sit upright adequately to see the projected eye chart. Possible undiagnosed glaucoma was excluded by determining the cup-to-disc (CTD) ratio of optic nerves through an undilated examination using slit-lamp biomicroscopy with a 90-diopter lens. In five patients, muscular weakness prevented adequate positioning for standard slit lamp biomicroscopy, and a portable slit lamp biomicroscope was utilized instead. We specifically looked for any signs of retinopathy such as microaneurysms, cotton-wool spots, retinal hemorrhages, exudates, and vascular changes. Signs of optic neuropathy, such as increasing cupping, paleness, and swelling, were also determined to rule out ophthalmic pathology.

### Outcome measurements

Retinal thickness was assessed by measuring the RNFL with SD-OCT (Spectralis; Heidelberg Engineering) using standard, commercially available software. The device used for all imaging obtained was the Spectralis OCT, HRA Camera FW Version 2.1.1.0, OCT Camera FW version 1.51.0.0, which produces highly precise measurements [28]. Software used for analysis of data was acquisition software version 5.4.7.0 by Heidelberg Engineering. All RNFL scans were obtained with a 12.0 degree circle diameter. We prioritized head stabilization, precise image alignment, and centration of the optic disc during the scan mode. No motion artifact was detected on any scan.

For each enrolled patient, RNFL thickness (mean and six sectors) was compared to mean values in the normative database built into the SD-OCT imager. This normative database is routinely used by ophthalmologists to evaluate abnormal changes in the optic nerve or retina during routine clinical visits to the ophthalmic clinic, and represents current standard of care. Normative values were provided by Heidelberg Engineering to enable us to perform independent statistical analyses, and were based on studies performed on healthy patients without ALS or ophthalmic diseases [29]. Moreover, the Spectralis normative values were age-adjusted to correspond to the age of enrolled ALS patients using a standard formula provided by Heidelberg Engineering. The formula provided to age-adjust normative database values was as follows:

age-adjustedpercentile=percentile+(subject’sageinyears-48.2)*(slope)

with different slope values provided by Heidelberg Engineering for mean RNFL as well as six sector RNFL values.

### Statistical analyses

The significance of the differences between the RNFL thickness values obtained from the patients in this study and corresponding age-adjusted normative values were assessed using the Wilcoxon signed rank test of median difference equal to zero. The relationships between RNFL thicknesses and other continuous variables (including visual acuity, intraocular pressure, ALSFRS-R score, ALSFRS-R progression rate, forced vital capacity (FVC), and CTD ratio) were assessed using linear regression. ALS disease severity was determined through the ALSFS-R scale in the ALS clinic; this value was analyzed for statistical correlation with RNFL thickness. All right eyes were analyzed as one population and all left eyes as a separate population, in order to mitigate for possible dependencies between eyes from the same individual. P-values less than 0.05 were considered significant. Analyses were carried out using SAS version 9.3 and JMP Pro 11. To ensure our study was adequately powered, the sample size needed for a 90% power study with a two-sided test for paired data with alpha = 0.05 was calculated using the mean differences in nerve fiber layer thickness detected in our study.

## Results

### Demographics

The demographics of our study population are summarized in Table 1, and highlight that only Caucasians and mostly male subjects were enrolled in our study, consistent with other ALS studies.

**Table 1:** Demographic characteristics of the study population.

	Study Subjects (n = 21)
Male	14 (66%)
Female	7 (34%)
Caucasian Race	21 (100%)
Age (Years)	59 (36–79)
ALSFRS-R Score	30 (2–40)
ALSFRS-R Progression at Enrollment	0.91 (0.1–4.2)
Forced Vital Capacity	65% (26%-92%)
Genotype Abnormalities in Genes Associated with ALS*	4 (19%)

Data are expressed as n (% total) for sex and race, and as mean (range) for age and ALSFRS-R Score. The data show a high number of Caucasian male ALS study patients, which is consistent with known ALS demographics.

*Specifically, 3 patients had pathogenic repeats in C9ORF72, and 1 patient had VUS in the DCTN1 and ALS2 genes.

### Ophthalmologic examination

Visual acuity, intraocular pressure, and average cup-to-disc ratio were assessed and summarized in Table 2 below. The results of all slit lamp examinations showed normal anterior segment findings, except for five patients who demonstrated mild nuclear sclerotic cataracts. However, these cataracts did not interfere with the quality of OCT signals. Overall, the participants in our study had good visual acuity, normal intraocular pressures, and no enlarged optic nerve head cupping.

**Table 2:** Ophthalmologic examination characteristics.

	Right Eye		Left Eye	
	Mean	Range	Mean	Range
Visual Acuity (Snellen)	20/30	20/20-20/50	20/30	20/20-20/50
Visual Acuity (LogMar)	0.19	0–0.4	0.14	0–0.4
Intraocular Pressure (mmHg)	14	8–18	14	8–19
Average Cup-to-Disc Ratio	0.3	0.1–0.6	0.3	0.1–0.6

Visual acuity was performed on 18 right eyes and 16 left eyes. Intraocular pressure was performed bilaterally on 19 subjects, and average cup-to-disc ratio was measured bilaterally on 16 subjects.

Because most ALS patients were unable to tolerate the additional 30 minutes required for dilating drops to take effect, undilated fundus examination was performed. Undilated posterior segment examination of study subjects revealed normal neurosensory retina and retinal blood vessels. Because systemic and retinal diseases such as severe diabetes typically would result in abnormal retinal blood vessels or intraretinal hemorrhages, our examination findings indicate that retinal pathologies that might affect OCT imaging were not present. Normal intraocular pressures, optic nerve CTD findings (Table 2), and healthy neuroretinal tissue indicate that our ALS subjects did not have advanced glaucoma or ischemic optic neuropathy, which can contribute to RNFL or macular thinning with OCT imaging.

### SD-OCT imaging

**Fig 1:**
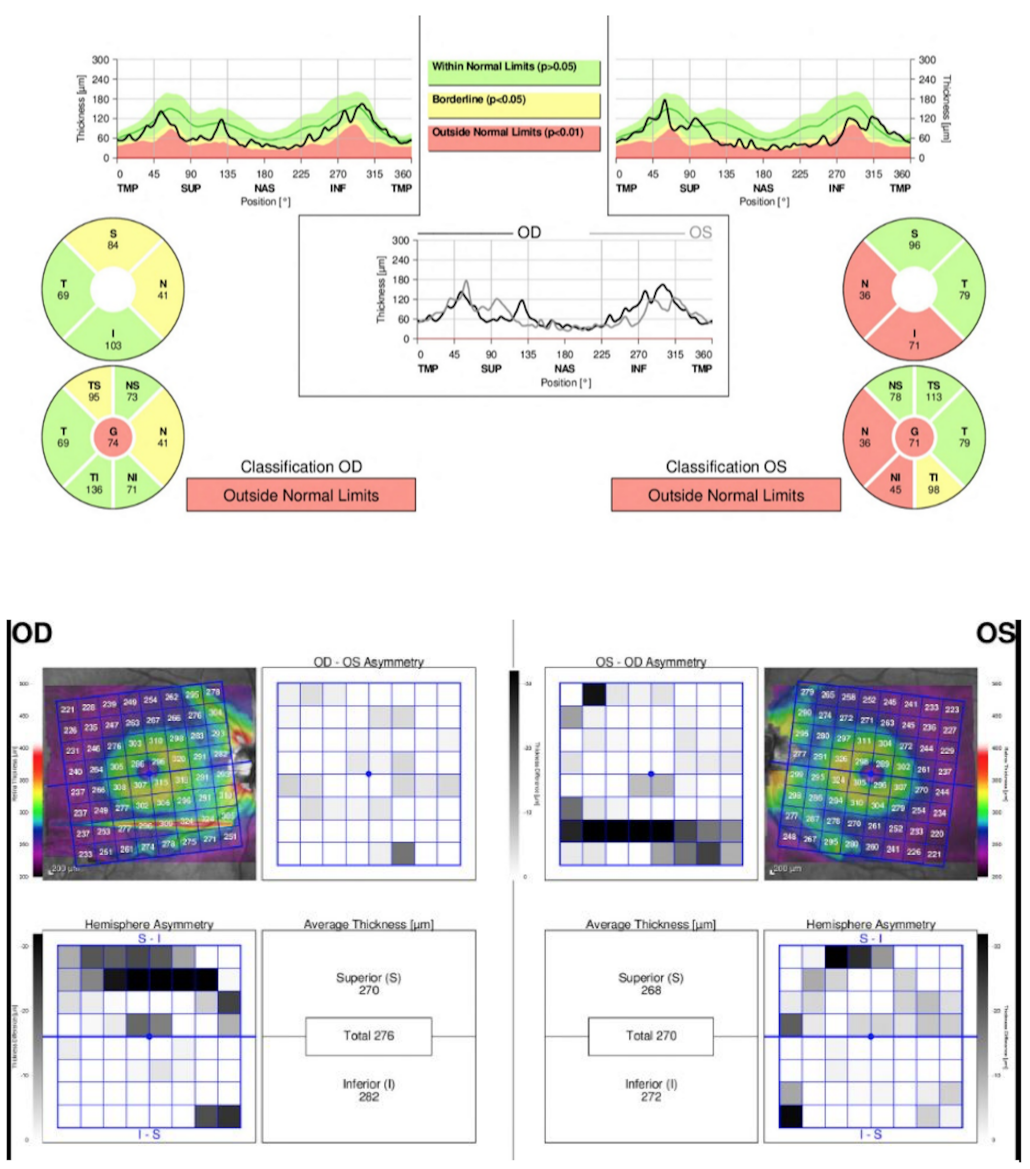
Representative clinical findings, SD-OCT and macular map from an ALS subject. OCT results were compared to the age-adjusted normative database by the integrated SD-OCT analysis software. Thin retinal areas are presented in a report format used in a typical clinic visit for ophthalmic examinations.

**Fig 2:**
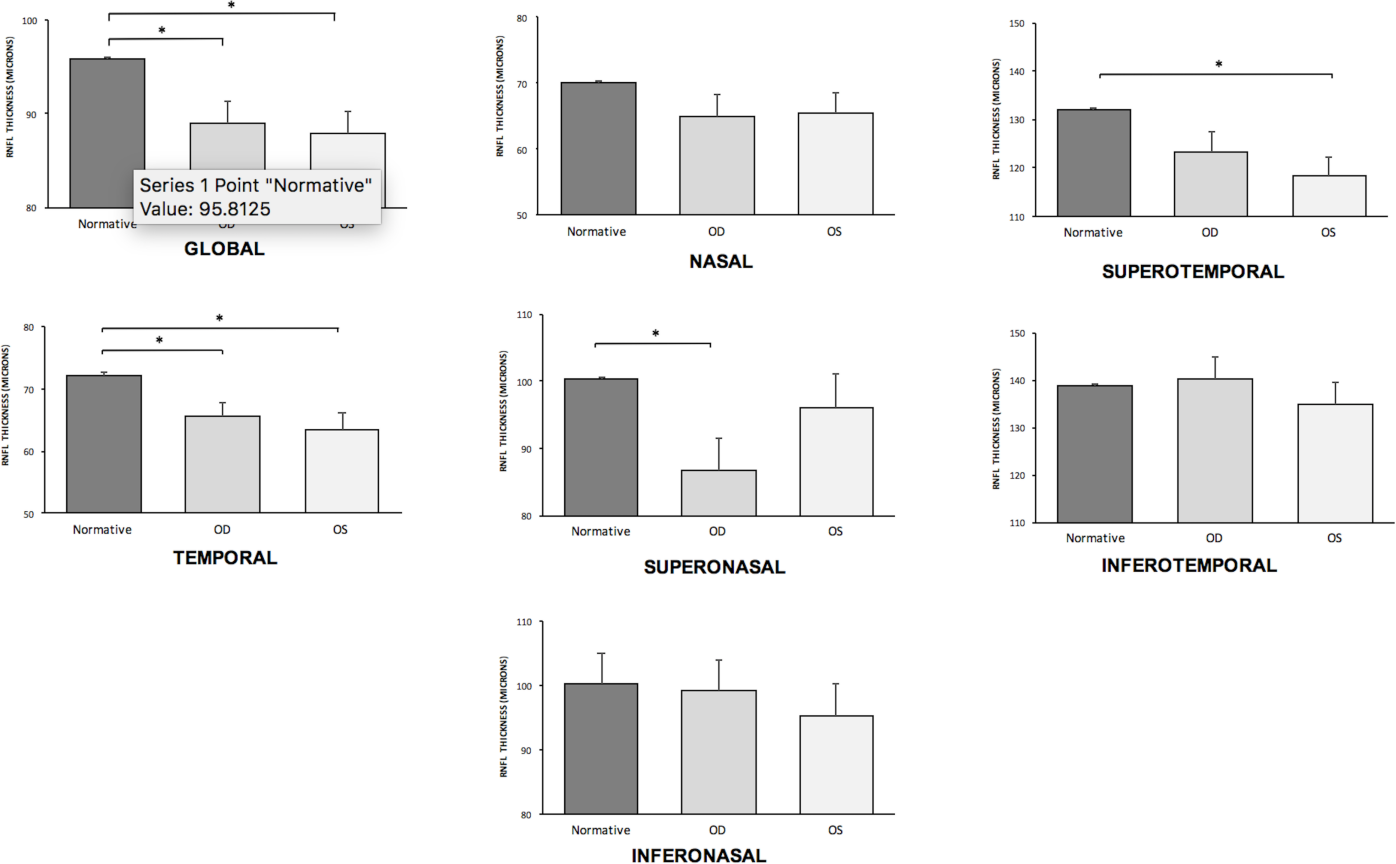
Mean and sectorial RNFL thickness comparison between ALS subjects and age-adjusted normal values. Total and six sector RNFL values were compared to the age-adjusted normal value utilized by the Spectralis SD-OCT system. The data presented here include mean ± standard error of the mean. Data for the right eye is indicated by OD and the left eye by OS. The right eye group showed significant thinning in total RNFL thickness, temporal sector RNFL thickness, and superonasal sector RNFL thickness. The left eye group had significant thinning in total RNFL thickness, temporal sector RNFL thickness, and superotemporal sector RNFL thickness.

SD-OCT imaging was successfully performed on all ALS study subjects despite positional challenges resulting from motor deficits. We include representative SD-OCT and macular map findings from one of the ALS subjects in our study (Fig 1). SD-OCT imaging of the 40 eyes of 21 patients included in the data analysis of this study displayed a mean RNFL thickness of 88.95 +/- 10.8 microns, significantly thinner than values in the normative database (95.81 +/- 0.8) (Table 3). The data showed RNFL thinning across the board when compared to normative values, and statistically significant thinning in nearly half of the six sector and global categories. Specifically, statistically significant thinning was found for mean total RNFL thickness and temporal thickness for both the right and the left eye, superonasal thickness for the right eye, and superotemporal thickness for the left eye, when compared to age-adjusted normative database values (Fig 2).

**Table 3:** Differences in RNFL thickness between ALS subjects and normative database.

	Right Eye n = 20			Left Eye n = 19		
RNFL Sector	Mean Difference (μm)	Standard Deviation (μm)	P-value	Mean Difference (μm)	Standard Deviation (μm)	P-value
Global	-6.86	11.03	0.043	-8.15	11.34	0.007
Temporal	-6.59	10.34	0.012	-9.75	12.11	0.003
Nasal	-6.55	14.49	n.s.	-6.13	14.38	n.s.
Superonasal	-15.2	20.97	<0.001	-6.16	23.34	n.s.
Inferonasal	-1.00	0.00	n.s.	-3.26	18.01	n.s.
Superotemporal	-8.63	18.78	n.s.	-13.9	18.41	0.007
Inferotemporal	1.47	20.78	n.s.	-4.36	22.41	n.s.

Mean and six sector RNFL thicknesses from the study subjects were compared to age-adjusted normal values to determine RNFL changes. Results are shown as differences; a negative value indicates RNFL thinning. There is an overall trend of RNFL thinning in all sectors and global mean. Statistically significant thinning is found in bilateral mean total RNFL thickness and temporal thickness, right superonasal thickness, and left superotemporal thickness. P-values were obtained with the Wilcoxon signed rank test of median difference equal to zero. n.s. = not significant.

### Correlation with ALS disease severity

**Fig 3:**
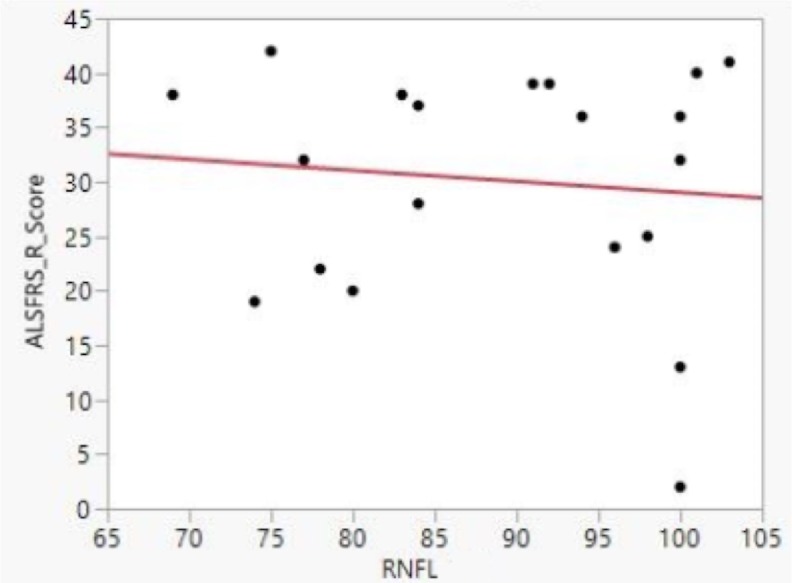
Bivariate fit for RNFL thickness and ALFRS-R score. No significant correlation between mean RNFL thickness and ALSFRS-R score was found. Please note that most ALSFRS-R scores were relatively high, indicating mild ALS disease severity. Data shown are from right eyes only, but we obtained the same result from left eyes: there was no correlation observed between ALSFRS-R and RNFL thinning.

We sought to determine whether severity of ALS symptoms correlated with OCT and ocular examination findings. ALS disease severity was assessed through the ALSFRS-R score, which is widely used in clinical studies [27]. Additionally, the ALSFRS-R progression rate and FVC were measured, and all three variables were analyzed against RNFL thickness. The ALSFRS-R score and progression rate, and the FVC for each patient were determined by a board-certified fellowship-trained neurologist with extensive experience in caring for ALS patients. After performing a bivariate fit between ALSFRS-R score and OCT data, no clear correlation between RNFL thinning and disease severity was observed in our patient cohort (Fig 3).

**Fig 4:**
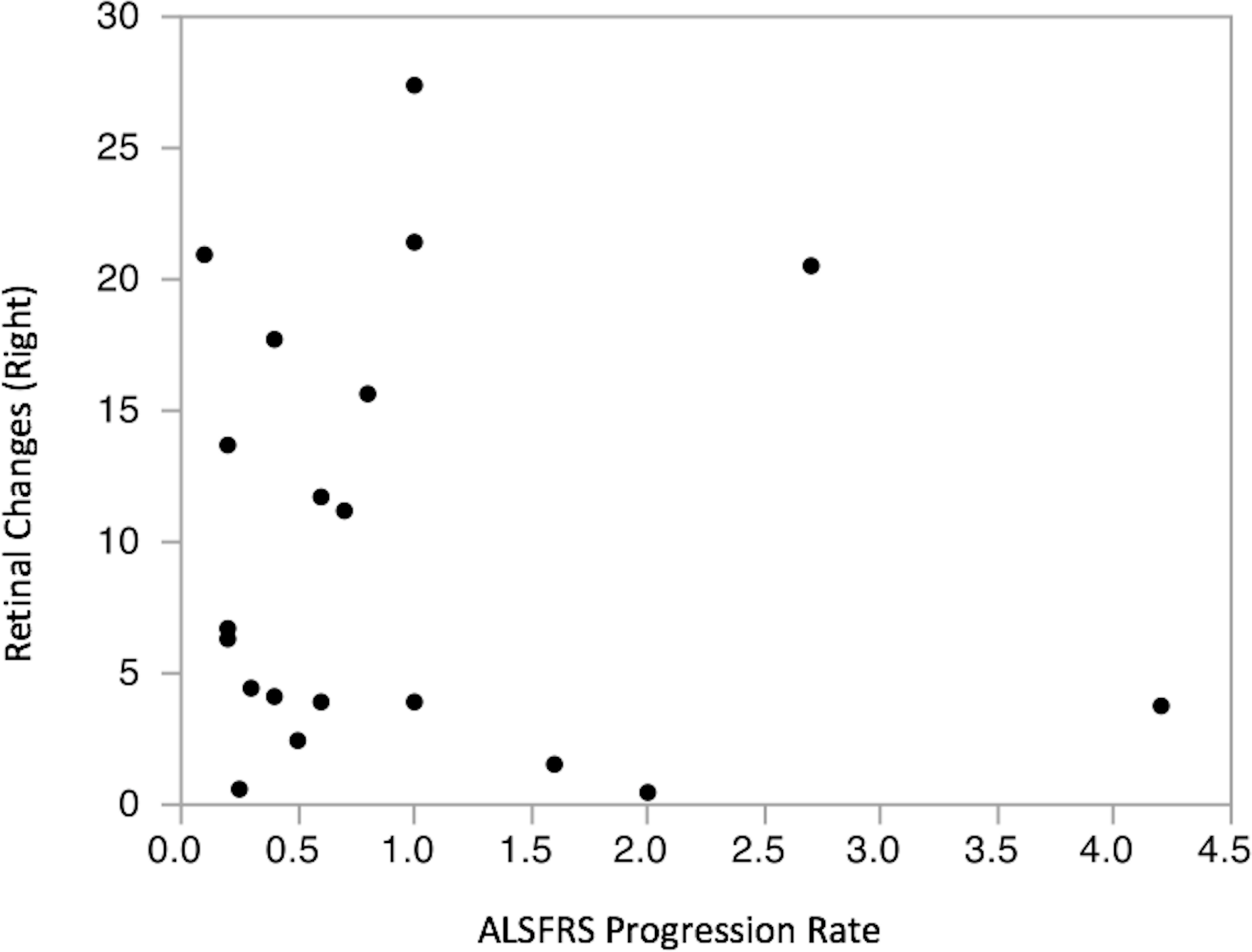
Bivariate fit for RNFL thickness and ALFRS-R progression rate. No significant correlation between mean RNFL thickness and ALSFRS-R progression rate was found. Again, data shown are from right eyes only, but the same result was obtained from left eyes.

A bivariate fit between ALSFRS-R progression rate and OCT data also showed no clear correlation (Fig 4).

**Fig 5:**
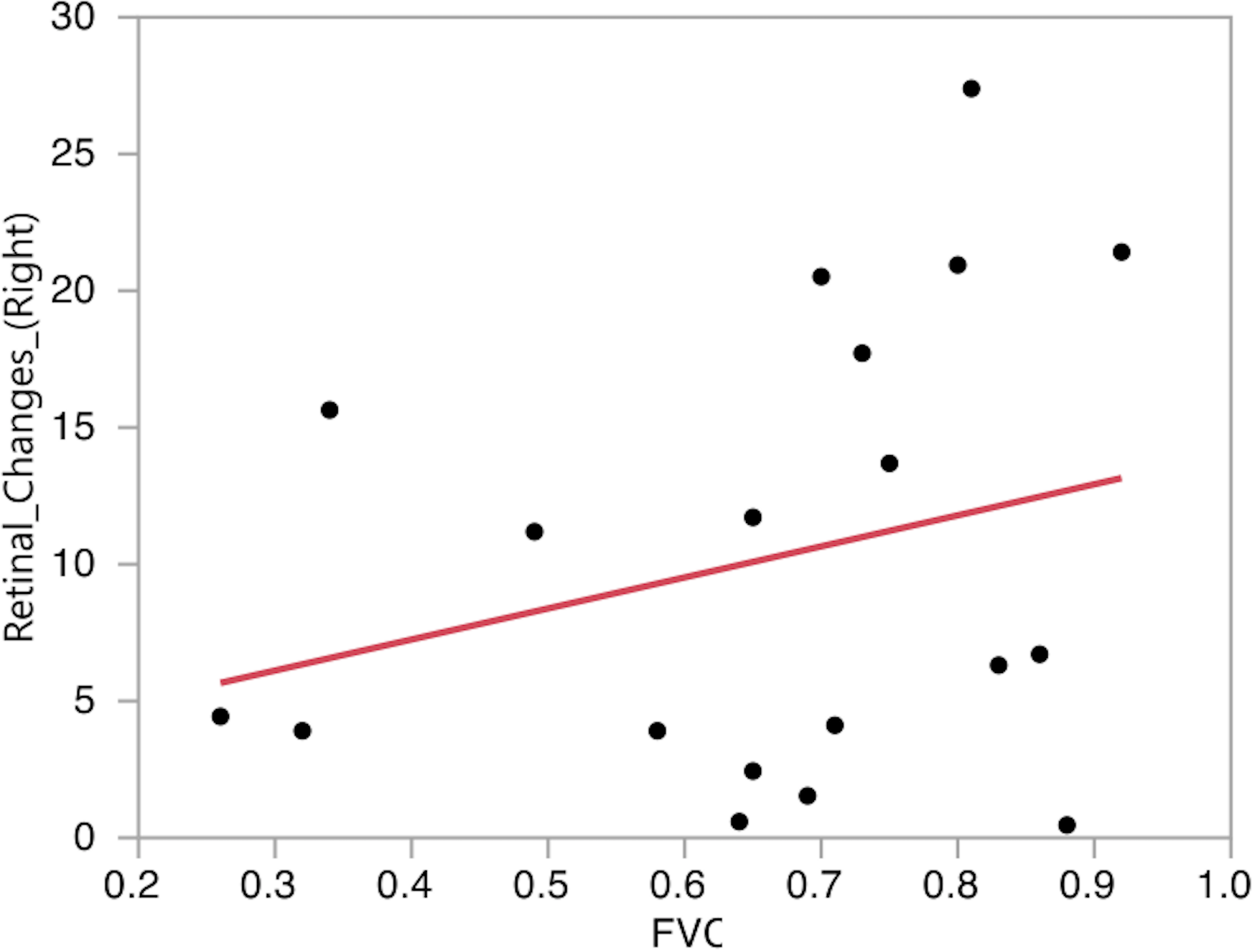
Bivariate fit for RNFL thickness and FVC. No significant correlation between mean RNFL thickness and FVC was found. The data shown are from right eyes only; the same result was found in left eyes.

Thirdly, a bivariate fit between FVC and OCT data showed no clear correlation (Fig 5).

**Fig 6:**
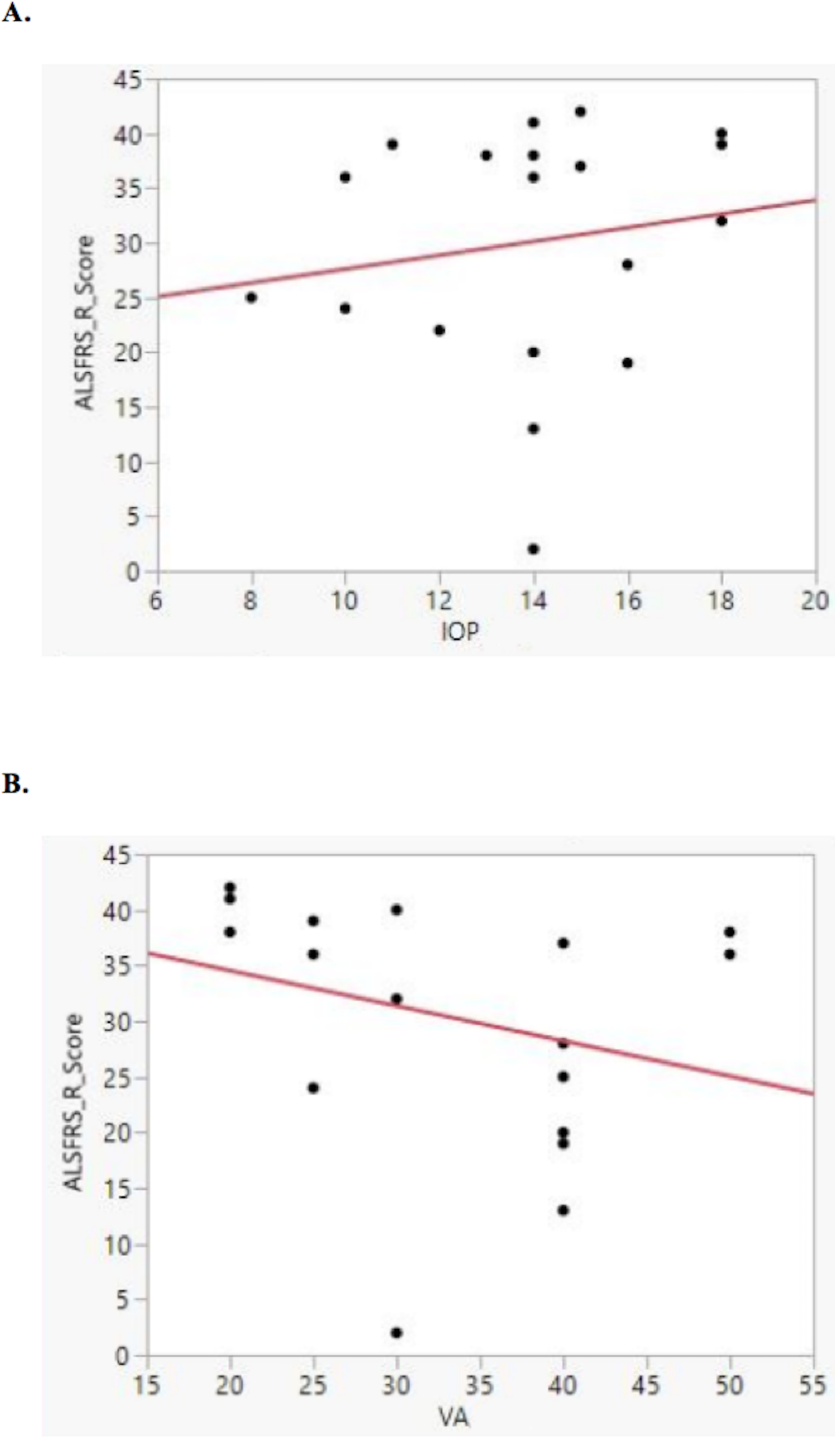
**Bivariate fit for ALFRS-R score and intraocular pressure (A), and ALFRS-R score and visual acuity (B).** (A) No significant correlation between ALSFRS-R score and intraocular pressure (IOP) were found. (B) Similarly, there was no correlation between ALSFRS-R score and visual acuity (VA). Data shown are from right eyes only, but analyses with left eye data were comparable.

Although patients with ophthalmic diseases were already excluded during the study recruitment process, an analysis was performed to determine if retinal thinning might be attributed to undiagnosed ocular conditions such as ocular hypertension or myopia. Using a bivariate fit, no correlation was identified between ALSFRS-R and visual acuity or intraocular pressure (Fig 6). However, taken together, our data analysis indicates that RNFL thinning is observed in ALS patients, and appears to be associated with ALS neurodegeneration rather than ophthalmic pathology or variations in ocular anatomy.

## Discussion

Our data supports our hypothesis that ALS patients without ophthalmic disease exhibit retinal thinning. Resultant data revealed a statistically significant RNFL thinning in the retinas of ALS patients when compared to the age-adjusted normative database utilized by the SD-OCT system and built-in software. Our approach of comparing data from ALS study patients to the normative values represent the gold standard of how standard OCT data are interpreted during a typical visit in the eye clinic. Because ophthalmic diseases that can impact retinal thickness were carefully excluded by detailed history and a comprehensive ophthalmic examination, retinal thinning in our study subjects is most likely to be associated with neurodegenerative pathophysiology inherent in ALS.

The inclusion of a detailed ophthalmologic examination of each ALS study patient represents an important methodological difference between this study and other reports that have examined the relationship between ALS and retinal thinning by OCT. In 2013, Roth *et al*. examined seventy-six ALS patients with OCT and found no significant difference between ALS patients and healthy controls [23]. In contrast, another study by Ringelstein *et al*. described RNFL and macular volume thinning in twenty patients with ALS compared to healthy controls [24]. However, in both studies, no detailed ophthalmic evaluation and exclusion for retinal diseases were reported. Numerous ocular and systemic medical conditions are known to be associated with retinal thinning that can be detected by SD-OCT imaging. In 2016, Hübers *et al*. excluded patients with a history of eye disease and found statistically significant RNFL thinning in seventy-one ALS patients compared to healthy controls [26]. However, exclusion of ophthalmic disease was only by history and no direct ophthalmic examinations were performed or reported to rule out eye diseases that were undiagnosed. Many ophthalmic diseases, such as glaucoma and diabetic retinopathy, are often undiagnosed unless patients have received routine annual eye examinations. In this context, our study excluded eye disease both by history and direct ophthalmic examinations by ophthalmologists trained to detect retinal and optic nerve disease. The resultant statistical analysis demonstrated no correlation between ALSFRS-R score with visual acuity, CTD ratio for the optic nerve, or intraocular pressure. Taken together, RNFL thinning detected through OCT imaging observed in our study is unlikely due to ophthalmic pathology.

Although our data showed statistically thinner RNFL in ALS patients, no correlation between ALSFRS-R score, ALSFRS-R progression rate, or FVC was detected when measured against retinal thinning. This might be due to several limitations of the study. Firstly, this was a small study which was powered to detect retinal thinning but a larger follow up study should be considered to correlate retinal changes to ALSFRS-R score, progression rate, or FVC. Second, correlation might only occur with specific ALS subtypes: in particular, ones with genetic associations shared with retinal ganglion cell loss in glaucoma (e.g. optineurin, TBK1, and ataxin2). Our study included genotyping, but had no participants with the above-mentioned genetic associations. A future study might include participants with glaucoma-associated ALS genotypes.

Instead of correlating retinal thinning with ALSFRS-R at a single time point, another approach can be utilized to correlate retinal findings with ALS disease severity and progression. A series of repeated OCT measurements made over several months or years would be more revealing. Furthermore, repeat retinal measurements could be normalized to the initial measurement (considered “baseline”) for comparison over time. Analysis of such a data set will reveal the extent and rate of retinal thinning, and correlate these findings to ALS disease progression. This approach may be more sensitive in correlating retinal changes to ALS disease severity and determining whether retinal changes can be utilized as an imaging biomarker for disease progression.

In conclusion, RNFL thinning in ALS patients that was not attributable to ophthalmic diseases was detected using SD-OCT imaging. Clinicians can consider adopting this rapid, non-invasive clinical instrument in an outpatient clinic as a tool to track disease progression in ALS patients. The development of smaller SD-OCT devices, including handheld systems [30–33], will facilitate retinal imaging of these severe ALS patients by overcoming logistical and medical challenges. Additionally, as newer imaging techniques such as DARC (detection of apoptosing retinal cells) become available, it may be possible to have real-time imaging of apoptosis in single neurons in the retina. Such advances may allow more precise measurements of neurodegenerative disease progression using the retina [11]. Further clarification of the temporal relationship between motor neuron degeneration and retinal neurodegeneration in ALS patients or animal models will aid in the use of the retina as a tool to monitor ALS disease progression and assess responses to potential therapies.

## Supporting information

S1 File ALS retinal study dataset. (XLSX)
